# Routine histopathology in total joint arthroplasty: clinical utility and economic evaluation from a tertiary care center

**DOI:** 10.1007/s11845-026-04333-y

**Published:** 2026-04-01

**Authors:** Arunava Kumar Bhuyan, Kiran Kumar Vedavyasa Acharya, Sachin Kumar

**Affiliations:** Department of Orthopaedics, Kasturba Medical College, Manipal Academy of Higher Education, Manipal, 576 104 Karnataka India

**Keywords:** Costs and cost analysis, Total knee arthroplasty, Total hip arthroplasty, Quality-adjusted life years

## Abstract

**Background:**

Routine histopathological evaluation of bone and synovium during total knee and hip arthroplasty (TKA and THA) is widely practiced in many institutions. However, its cost-effectiveness, particularly in resource-limited settings, remains unclear. This study aims to assess whether such routine analysis offers clinical value commensurate with its cost.

**Materials and methods:**

We retrospectively analysed 1027 consecutive primary total joint arthroplasty (823 knees and 204 hips) and compared our presumed preoperative diagnosis with the histopathological diagnosis made by the pathologists. Intraoperatively, femoral head specimen and bone pieces along with synovium removed during taking bone cuts were collected for THA and TKA respectively. The specimen was fixed with 10% neutral buffer formalin, treated with paraffin and cut with microtome. Staining was done with standard hematoxylin and eosin red stains. The cases were then grouped as concordant (matching), discrepant (not matching but no change in patient management) and discordant (not matching and with change in patient management). The cost-effectiveness analysis was conducted using the incremental cost-utility ratio (ICUR) framework, where cost-effectiveness was defined as < INR 4,82,472 ($5,597) per QALY gained.

**Results:**

Of the 1027 consecutive cases, 883 (86%) were concordant, 144 (14%) were discrepant and none were discordant. Total expenditure for histopathological analysis over 10 years amounted to Rs. 22,59,300 ($26,191) with 0 QALYs gained. No malignancies or unexpected diagnosis altered patient management.

**Conclusion:**

Routine histopathological analysis from primary TKA and THA did not yield clinically actionable findings and was not cost-effective in our setting. The findings support selective use of histopathology based on clinical suspicion.

## Background

Total joint arthroplasty surgeries of hip (THA) and knee (TKA) are among the most frequently performed surgeries worldwide in the field of orthopaedics and are projected to grow exponentially worldwide [1, 2]. Policies to send routine histopathological samples have been introduced for completion of diagnosis and avoiding medicolegal issues [3]. Whereas some patients have benefited from such routine testing, most of the literature cannot justify such screening to the society [4, 5].

Due to the lack of robust literature pertaining to southwestern India with emphasis on the nuances of histopathological analysis and the related cost-effectiveness of such evaluation, there remains a knowledge gap from a clinical and economic perspective. Thus, our study aimed to evaluate the histopathological analysis of bone and synovium obtained during these surgeries and to do a cost-effectiveness analysis of such routine testing.

To address these objectives, our retrospective analysis utilized the ICUR formula, a method recommended by the WHO for assessing the cost-effectiveness of healthcare interventions [6]. The significance of such a study would address the need for such testing and potentially help save money in an economically developing nation such as India.

## Materials and methods

**Fig. 1:**
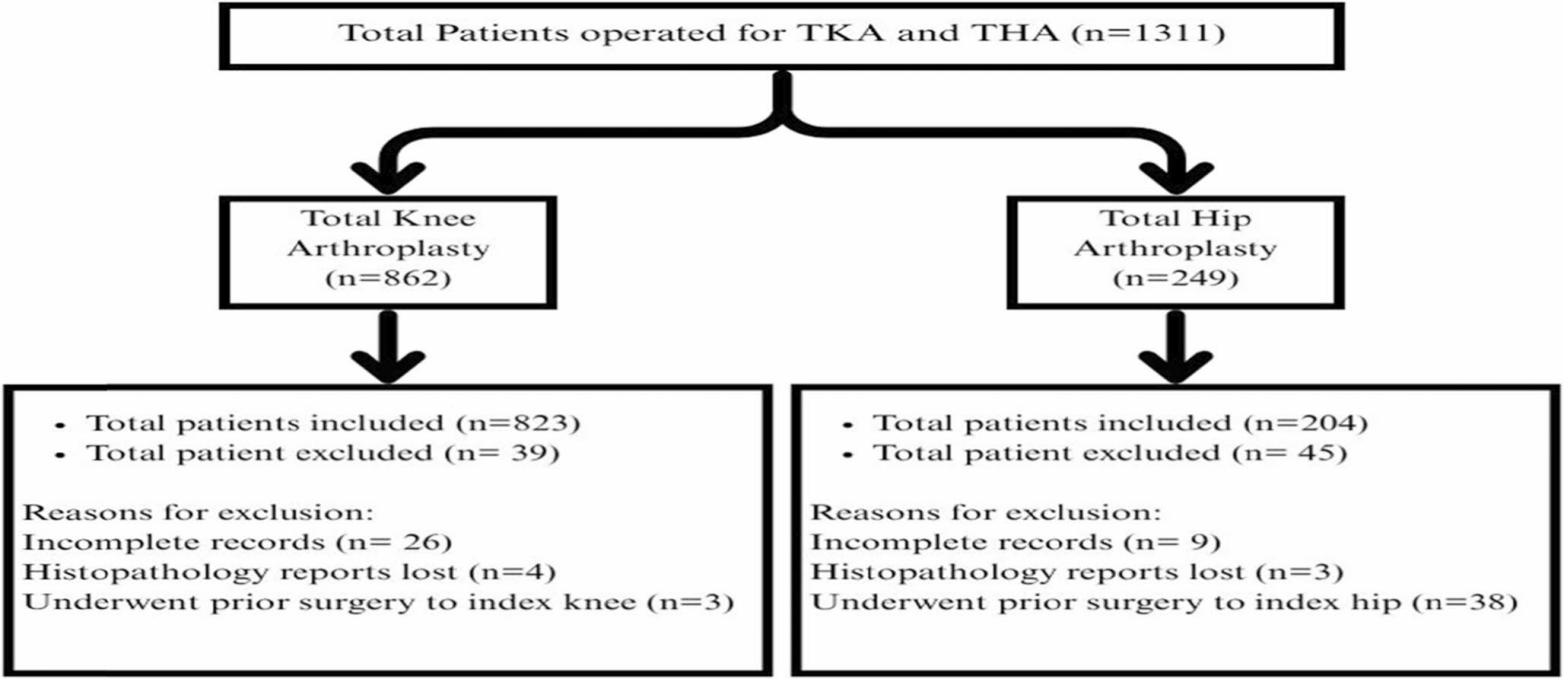
Flowchart summarizing patient included in final analysis

Institutional Ethics Committee approval and Clinical Trials Registry of India registration was done, and we performed a retrospective review of our institution’s primary elective hip and knee total joint arthroplasty register between January 2012 through December 2022. We encountered 1311 consecutive total knee and total hip arthroplasties done in that period. After excluding the cases which had undergone any surgical intervention to the same hip or knee in the past or with incomplete records as summarized in Fig. 1, we identified 1027 cases of total joint arthroplasty making the sum of 823 total knee arthroplasty and 204 total hip arthroplasty which had complete records and did not undergo any prior surgery to the index site. The method of collection of samples was standard across the institute. After opening of the knee joint, the surgeon inspected the articular cartilage and synovium. While taking the distal femoral cuts and tibial cuts, the bone and synovium are collected. In the hip joint, the femoral head is extracted in-toto after the neck cut.

### Histopathological analysis

From the primary total knee arthroplasty group, both femoral and tibial resection samples along with synovium (wherever clinically indicated) were obtained while from the primary total hip arthroplasty group resected femoral head was obtained using standard steps of surgery. The bone specimen was treated with 10% neutral buffer formalin to preserve cellular and tissue architecture. The fixed synovial samples are then processed routinely for paraffin embedding: they are dehydrated through graded alcohols, cleared in an intermediate solvent (xylene) and infiltrated with molten paraffin wax using an automated tissue processor, ensuring complete penetration of the paraffin medium into the tissue. Similarly, bone specimens undergo decalcification using acid solutions (nitric acid)/chelating agents with careful monitoring for the complete removal of mineral content, as the dense mineralization prevents adequate thin-section cutting. Once decalcification is complete, the bone is dehydrated, cleared, and infiltrated with paraffin using the same protocols applied to soft tissue. Both processed bone and synovial tissue are then embedded in paraffin blocks with careful orientation to ensure the most diagnostically relevant areas are positioned in the cutting plane. Thin serial sections of 3–5 micrometre thickness are subsequently cut using a rotary microtome and mounted on glass slides, where they are stained with haematoxylin and eosin (H&E) for routine histological evaluation. The pathologist interpreted 823 total knee samples and 204 total hip samples. The pathologist interpreted the pathological diagnosis based on both gross and microscopic characteristics. Confirmatory tests such as immunohistochemistry analyses and/or flow cytometry were performed on the discretion of the pathologist; however, analysis of such testing is beyond the scope of our study and has been omitted from our reporting.

After these intraoperative histopathologic samples were analysed, we compared these to the presumed preoperative diagnosis and categorized the cases into 3 categories: A concordant result, defined as agreement between presumed clinical diagnosis and histopathological diagnosis. A discrepant result, defined as disagreement between presumed clinical diagnosis and histopathological diagnosis that should not be expected to alter patient management and a discordant result, defined as disagreement between presumed clinical diagnosis and histopathological diagnosis that should be expected to alter patient management.

### Cost-effectiveness analysis

The total cumulative cost of histopathological analysis of samples sent during primary THA and TKA was obtained for the period 2012–2022 using rates according to institutional financial department for 2022 adjusted Indian Rupees. This resulted in a cost of Rs. 2200 per histopathological analysis (This excluded the cost of further investigations that was based on the histopathological analysis like immunohistochemistry or flow cytometry). Therefore, we only report the cost of identifying a discordant case. This cost was calculated by identifying the number of cases in each category, then dividing it by the total number of histopathological analyses performed. This is a standard set by numerous studies as described in our extensive review of literature.

We utilized the “incremental cost utility ratio (ICUR)” approach to perform the cost-effectiveness analysis [6]. The ICUR is calculated by dividing the overall expense of histopathologic examination by the ‘quality adjusted life years (QALYs)’ gained by patients who underwent this screening [7]. ICUR acts as a metric to evaluate the worth of healthcare spending, measuring both the quantity and quality of life years saved due to the intervention. The World Health Organization (WHO) sets the benchmark for evaluating the cost-effectiveness of interventions as an Incremental Cost Utility Ratio (ICUR) that does not exceed three times a nation’s Gross Domestic Product (GDP) [6]. Interventions that are below this limit are considered cost-effective.

As per the World Bank Open Release, the average GDP per capita in India from 2012 to 2022 was Rs. 1,60,824 [8]. This implies that interventions costing less than Rs. 4,82,472 per QALY gained are regarded as cost-effective.

## Results

### Demographic details

In our retrospective analysis, we found that the mean age of the primary total knee arthroplasty group was 64 years while the total hip arthroplasty group was significantly younger at 51 years. There was a preponderance among women in the knee group while men underwent total hip arthroplasty more in the period. Patient demographics of 1027 cases are summarized in Table 1.

**Table 1:** The demographic details of the patients in the primary TKA and primary THA groups

Particulars	Primary TKA	Primary THA
Number of cases	823	204
Age in years (Mean + Standard deviation)	64.42 ± 8.28	51.17 ± 14.94
Sex ratio (Women: Men)	591:232 (2.5:1)	124:80 (1.5:1)

### Preoperative diagnosis

For the study, the retrospective analysis of our register revealed the most common diagnoses to be quite different for both the primary total knee arthroplasty and primary hip arthroplasty groups. Primary osteoarthritis of the knee joint (92%) was the most encountered diagnosis encountered in the knee group, the data is quite different thereafter. In the primary total knee arthroplasty group, rheumatoid arthritis (7.5%) and post traumatic secondary arthritis (< 1%) were the next encountered diagnosis. In contrast, the primary total hip arthroplasty group, avascular necrosis (33%) and primary osteoarthritis of hip joint (32%) were the most encountered diagnosis. Subsequently, the cases we encountered were sporadic with post-traumatic secondary arthritis, secondary acetabular dysplasia, rheumatoid arthritis, ankylosing spondylitis, suspected secondary metastatic deposit of femoral head, neglected Perthes disease, ochronotic arthritis and old treated tubercular arthritis of hip joint. These above-mentioned findings have been highlighted in Tables 2 and 3 respectively.

**Table 2:** The preoperative clinical diagnoses for primary TKA group

Parameters	Frequency	Percentage (%)
Primary Osteoarthritis	757	92
Rheumatoid Arthritis	62	7.5
Post traumatic secondary arthritis Pigmented villonodular synovitis	3 1	< 1 < 1

**Table 3:** The preoperative clinical diagnosis for primary THA group

Clinic-radiological diagnosis	Frequency	Percentage (%)
Avascular necrosis	67	33
Primary Osteoarthritis	66	32
Post Traumatic Arthritis	15	7
Ankylosing spondylitis	12	5
Acetabular dysplasia	11	5
Secondary Degenerative Osteoarthritis	10	5
Rheumatoid arthritis	9	4.5
Neck of femur fracture	7	3.4
Secondary metastasis	2	< 1
Tubercular arthritis	2	< 1
Osteochondroma	1	< 1
Ochronotic arthritis	1	< 1
Neglected Perthes Disease	1	< 1

It must be noted that when we used the term primary osteoarthritis, we mean to express articular degeneration without any apparent underlying reason at the time of preoperative clinical evaluation.

The femoral and tibial bone resection specimen that was analysed by the pathologists are represented in Table 4, degenerative joint disease was the most common diagnosed condition in the knee (90%) Inflammatory arthritis such as rheumatoid arthritis and other entities was more frequently encountered in the knee and were represented as either rheumatoid arthritis/inflammatory arthritis (3%) or as synovitis (5%) in the histopathological analysis of bone and synovium. We also encountered 5 cases calcium pyrophosphate deposition disease, 3 cases of avascular necrosis of the knee joint and 1 case of osteochondroma of the knee joint.

**Table 4:** Histopathological diagnosis for primary TKA group

Parameter	Knee Group(number of cases)
Degenerative joint disease	746
Synovitis	40
Rheumatoid arthritis/Inflammatory arthritis	24
Calcium pyrophosphate deposition disease	5
Avascular Necrosis	3
Osteochondroma	1
No Evidence of disease	10

Femoral head resection specimen histopathological analysis (Table 5) revealed degenerative joint disease hip (56%) as the most common followed by avascular necrosis (20%). 8 cases had consistent fracture changes on microscopic examination. We also encountered 11 cases of inflammatory arthritis which include 6 cases of nonspecific synovitis and 5 cases of inflammatory arthritis including ankylosing spondylitis and rheumatic arthritis on histopathological evaluation. This was followed by 1 case each of metastatic deposit, osteochondroma of the hip joint, ochronotic arthritis and Perthes disease.

**Table 5:** Histopathological diagnosis for primary THA group

Parameter	Hip Group(number of cases)
Degenerative joint disease	116
Avascular necrosis	42
Fracture	8
Synovitis	6
Inflammatory arthritis	5
Metastatic deposits	1
Perthes disease possibility	1
Ochronotic arthritis	1
Osteochondroma	1
No evidence of disease	23

No definitive histopathological diagnosis could be reached from 10 samples of the knee joint and 23 samples of the hip joint and were reported as “No evidence of disease”. We have decided not to exclude these from our study as it provides a real-world representation of what could be expected from such routine testing.

### Cost effectiveness analysis

For the 823 primary total knee arthroplasty cases, the preoperative clinic-radiological diagnosis matches with the intraoperative histopathological analysis in 749 cases and was grouped as “Concordant”. In 74 cases, the clinic-radiological diagnosis was different from the intraoperative histopathological analysis however, the histopathological analysis did not lead to any significant change in the further management of the patient and these cases were grouped as “Discrepant”.We encountered no cases where the preoperative clinic-radiological diagnosis differed significantly from the histopathological analysis reports which would warrant a need for change in management protocols of the patient. Hence, we encountered no “Discordant” cases in the knee group.

In the primary total hip arthroplasty patients, with the above-mentioned definitions, we encountered 134 concordant cases, 70 discrepant cases and no discordant cases. The findings are summarized in Table 6.

**Table 6:** Summary of diagnostic consistencies by histopathology with preoperative reports

Parameter	Knee *n*/ (%)	Hip *n*/ (%)
Concordant	749 (91)	134 (66)
Discrepant	74 (9)	70 (34)
Discordant	0	0

Using standard ICUR formula and cut offs, between the period 2012–2022, Rs. 22,59,300 was used for routine histopathological analysis for a combined total knee and hip arthroplasty surgeries to encounter no discordant findings and loss of 0 QALYs. This is represented in Table 7. Individually, the samples sent from routine total knee arthroplasty surgeries contributed to Rs. 18,1,500 with no discordant findings. The samples sent from routine total hip arthroplasty surgeries contributed to Rs. 4,48,800 with no discordant findings.

**Table 7:** Cost of histopathological analysis

Parameter	Cost of histopathology (in Rupees)
Primary TKA	18,10,500
Primary THA	4,48,800
Overall	22,59,300

## Discussion

Routine testing policies such as routine histopathological analysis of bone and synovium from every primary total knee and hip arthroplasty surgery have come into practice due to a multitude of well-intentioned reasons by providing an opportunity to screen for potentially life-threatening disease, avoid medicolegal implications arising out of such rare cases. Compared to the past, the average age of patients undergoing total joint arthroplasty is at a decline [2, 9]. In our study, we encountered an average age of 64 years and 51 years, respectively for primary TKA and THA, which has decreased from 65 to 75 years age group in the past [10, 11]. This increase in the number of patients undergoing total joint arthroplasty is most likely due to increased access to healthcare and orthopaedic infrastructure. Thus, the surgeon must bear in mind that when every effort is being made to offer total joint arthroplasty surgery to the general populace, the clinical value of such routine testing should be weighed against the cost. Whereas multiple studies have been conducted worldwide regarding the cost-effectiveness of such routine histopathological analysis and found not to be cost-efficient, hence it can be safely omitted. A very strong argument can be made that the cost of routine histopathological analysis does not outweigh the cost of life-altering treatment, if said diagnosis can be reached by a simple screening tool. While literature states that only 1 in 425 to 770 femoral heads [12] and 1 in 3200 knee [5] specimens turn out to be positive for previously undiagnosed malignancies, the cost is justified to the patients. However, the justification to society remains to be addressed to distribute equitably the already scarce health care resources.

In the primary TKA group, we encountered histopathological analyses which were in concordance with preoperative clinical diagnosis in 92% cases and 8% discrepancies were noted. We encountered few newly diagnosed cases of osteochondroma and calcium pyrophosphate disease. However, due to the benign nature of these conditions and no change in management postoperatively, these cases were classified as discrepancies [13]. While the primary THA group showed more variation in histopathological analysis, 66% of these were concordant and 34% were discrepant. We encountered 1 case of metastatic deposits, but the patient was a known case of metastasis, and such histopathological analysis did not alter the further management of the patient. In our 10-year retrospective group, we countered 32% cases of primary osteoarthritis of the hip, which is significantly more than the standard literature. However, a similar 5-year retrospective study in North India found 14.4% prevalence [14]. Coastal Karnataka is a hot-bed for Handi-Godu disease, a form of endemic primary osteoarthritis affecting the knees and hips. Our records also showed a higher prevalence of avascular necrosis of the hip joint; this can be explained due to the lack of specialized orthopaedic services in peripheral centers, hence the early diagnosis of avascular necrosis prior to collapse of femoral head is not possible. Our institution is a tertiary referral center which caters to a significant population of the region. These patients present rather late and hence undergo Total hip Arthroplasty. We did not encounter any discordant cases in either group.

In accordance with the previously mentioned ICUR formula, a total of Rs. 22,59,300 was utilized collectively in primary THA and TKA for histopathological analysis with no discordant results and 0 QALYs gained. However, individually Rs. 18,10,500 and Rs. 4,48,800 were utilized by the primary TKA and THA groups respectively. As the overall cost of histopathological analysis was more than the ICUR cut-off, such routine testing is inferred as not cost-effective screening tool.

The results of this study align closely with existing research on diagnostic discrepancies and cost thresholds. Cormier et al. (2020) found that such routine evaluation can inculcate a cost between Can$ 250,000 to Can$ 350,000 (INR 1.5 Cr to 2.2 Cr) annually [15]. While Koss et al. (2021) reported cost per discordant finding to be above $ 371,000 (INR 3.2 Cr) and was thus not cost-effective [16]. However, a study by DiCarlo et al. (2014) stated that, although such routine evaluation is not cost-effective, such testing only amounts to 0.5% of the total healthcare expenditure for total joint arthroplasty. This poses a dilemma whether we want to risk eliminating 100% quality assurance to save 0.5% cost^17^. On the other hand studies by Zwitser et al., Anibas et al. and Liow et al. have advocated for the routine testing as it has potential utility for early detection, prompt intervention and in turn extend life expectancy of the patient who are diagnosed with life-altering diagnoses such as Waldenstrom macroglobulinemia, B-Cell Lymphoma or other occult malignancies [12, 18, 19].

However, in the past 2 years a change in trend is noticed to a risk-stratified, selective approach based on clinical judgment which may be appropriate for specific populations, particularly femoral neck fracture patients where the incidence of incidental metastatic disease is demonstrably higher. Birsel et al. (2025) noted a discrepancy rate of 2.48% with respect to incidental diagnosis of metastatic bone disease for femoral neck fracture groups while the elective hip replacement group had 0% [20]. DiCarlo et al. noted a higher discrepancy rate due to better understanding of subarticular insufficiency fractures [17]. Although, both studies could not justify the cost-effectiveness of such routine testing and the time required to process and study orthopedic tissue, the argument they put forward is if an institution is willing to compromise on 100% quality assurance in order to incur savings of ~ 0.5% of the total hospital expenditure20. However, a drawback of such a generalized dictum is that it would hardly translate to the developing economies such as India where our study is conducted where in major institutions primary total hip and knee surgeries are conducted under multiple public welfare schemes where reimbursement policies tend to be stringent. No study of similar nature is conducted in this part of the world in the recent years which could adequately address these issues.

While the study was retrospective, the findings provide practical recommendations for the selective use of histopathology in routine arthroplasty. An endeavor could be undertaken to incorporate artificial intelligence and machine learning in the prediction for histopathological analysis based on preoperative patient-specific factors.

## Limitations of the study

The retrospective single-center design may limit generalizability to other populations and healthcare systems. Exclusion of patients with prior surgery to the index joint reduced the representation of post-traumatic secondary arthritis cases. Additionally, while no discordant cases were found, the zero QALY gain estimate may underestimate potential long-term benefits from detecting certain diagnoses in a larger sample. Prospective multicenter studies with larger cohorts and integration of advanced predictive tools could validate and refine these conclusions.

## Conclusion

This 10-year retrospective study found that routine histopathological analysis in primary total knee and hip arthroplasty cases did not identify any discordant diagnoses that altered patient management. The cost-effectiveness analysis demonstrated that the substantial expenditures on routine histopathology did not yield measurable quality-adjusted life years (QALYs), indicating it is not cost-effective in this setting. These findings support a selective rather than routine approach to histopathological evaluation in primary arthroplasty to improve resource allocation without compromising patient care.

## Abbreviations

TKA: Total Knee Arthroplasty
THA: Total Hip Arthroplasty
QALY: Quality adjusted life years
ICUR: Incremental Cost–Utility Ratio
Can$: Canadian Dollars
$: United States Dollars
INR: Indian Rupees

## Declarations

### Disclose generative AI use

Not used.

### Clinical trial number

CTRI/2024/02/079170 ( Registered with Clinical Trials Registry – India on 24/02/2024).
